# A shift in circulating rotaviral genotypes among hospitalized neonates

**DOI:** 10.1038/s41598-022-06506-y

**Published:** 2022-02-18

**Authors:** Sudhabharathi Reju, Padma Srikanth, Sribal Selvarajan, Reuben Kuruvilla Thomas, Ramya Barani, Prakash Amboiram, Gunasekaran Palani, Gagandeep Kang

**Affiliations:** 1 Department of Microbiology, Sri Ramachandra Institute of Higher Education and Research, Chennai, Tamil Nadu India; 2 Department of Neonatology, Sri Ramachandra Institute of Higher Education and Research, Chennai, Tamil Nadu India; 3 Former Director, King Institute of Preventive Medicine and Research, Chennai, Tamil Nadu India; 4 Wellcome Trust Research Laboratory, Christian Medical College, Vellore, Tamil Nadu India

**Keywords:** Infectious diseases, Microbiology, Virology, Paediatrics, Public health

## Abstract

In neonates, rotavirus (RV) infection is generally nosocomial. The control of rotaviral infection within hospital settings is challenging due to prolonged shedding of the virus and contamination of the surrounding environment. There are few studies that have reported asymptomatic infection within neonates. In this study, neonates were screened for RV infection and possible clinical manifestations that may play a role in RV acquisition were analysed. Stool samples were collected from 523 hospitalized neonates admitted for > 48 h in a low-cost and higher-cost tertiary centre. RV antigen was screened using ELISA and the samples which tested positive were confirmed by semi-nested RT-PCR. RV was detected in 34% of participants and genotypes identified included G12P[11] (44.4%), G10 P[11] (42.6%), G10G12P[11] (10.1%) and G3P[8] (2.9%). ICU admissions were associated with higher viral shedding (p < 0.05). Hospitalization in the low-cost facility ICU was associated with higher RV acquisition risk (p < 0.05). RV was detected in higher rates (36.9%) among neonates with gastrointestinal manifestations. G10P[11] was the predominant genotype for several years (1988–2016) among neonates within India. The preponderance of an emerging G12P[11] genotype and heterotypic distribution was documented. RV surveillance is important to identify emerging strains and establish the road ahead in managing RV infection.

## Introduction

Globally, rotavirus (RV) has been one of the major health concerns in children under five years of age. RV was responsible for approximately 258 million episodes of acute gastroenteritis (AGE) and 128,500 deaths worldwide in 2016^1^. In 2013, there were 47,100 RV-related deaths in India that contributed to 22% of the global burden^2^.

RV belongs to the family *Reoviridae*. The genome is comprised of eleven segments of double-stranded RNA that encode for six structural and six non-structural proteins^3^. RV infection manifests in a varied spectrum of illnesses in humans ranging from mild asymptomatic to severe symptomatic illnesses. RV infections in neonates are usually mild or asymptomatic^4^. In neonates, RV infection rarely causes GI symptoms such as diarrhea, vomiting, dehydration, necrotizing enterocolitis (NEC), abdominal distention, feeding intolerance, hematochezia and gastroesophageal reflux^5^. Interestingly, the circulating RV strains in neonates differ from the circulating strains in older infants and adults.

There is a paucity of data on RV infection among neonates with GI and non-GI manifestations^5^. Also, the link between genotype and clinical manifestation has not been well established. This study focuses on the identification of the co-circulating RV strains and to assess the associated risk factors among neonates.

## Materials and methods

### Participants: neonates (< 28days)

The study was approved by the Institutional Ethics Committee of Sri Ramachandra Institute of Higher Education and Research adhering to the declaration of Helsinki, 1964. Neonates who were admitted in the neonatal intensive care unit (NICU) and neonatal nursery of Sri Ramachandra Medical centre (higher-cost facility) and Sri Ramachandra Hospital (low-cost facility), Chennai, India were included in this study. Informed consent was obtained from the parents/guardian prior to enrolment in the study. Only neonates who were admitted > 48h in the neonatal nursery/ICU with or without gastrointestinal symptoms were included in the study.

A proforma was used to obtain demographic details and clinical presentation such as gastrointestinal (GI) manifestations. GI manifestations such as diarrhea, vomiting, abdominal distension, feeding intolerance, necrotizing enterocolitis, gastroesophageal reflux, neonatal cholestasis were considered symptomatic. Non-gastrointestinal (non-GI) manifestations such as respiratory distress syndrome, transient tachypnoea, apnoea of prematurity, hypoglycaemia, hyponatremia, hyperbilirubinemia, seizure, sepsis were considered asymptomatic with regard to RV infection^5^.

### Study design and period

This is a cross sectional study conducted between September 2016 and November 2019. A total of 523 stool samples were collected from neonates (< 28 days old) in the neonatal ICU and neonatal nursery.

### Sample collection and transportation

Stool samples were collected in sterile containers, transported in cold chain to the laboratory within 2 h, processed and stored at − 80 °C. Screening of RV antigen was performed using Enzyme Linked Immunosorbent Assay (ELISA) (Premier™ Rotaclone^®^ Meridian Bio-science Inc. Cincinnati, USA). The assay was performed as per manufacturer’s instructions. Samples with an OD value of > 0.15 were considered positive. Samples that tested positive for RV were genotyped by VP7 and VP4 semi-nested RT-PCR. Positive controls (G1P[8], G2P[4], G12P[8]) were procured from Wellcome Trust Research Laboratory, Christian Medical College, Vellore, India.

### Extraction of RNA

RNA was extracted from 20% faecal suspension via spin column-based extraction (QIAamp Viral RNA Mini kit, Germany, Qiagen) as per kit protocol. Complementary DNA (cDNA) was generated by reverse transcription in the presence of random primers (Invitrogen, USA), using reverse transcriptase enzyme, MMLV-RT-Moloney Murine Leukemia Reverse Transcriptase (Invitrogen, USA)^6^.

### Genotype identification

The cDNA obtained from the extracted RNA was used as a template for the VP7 first round PCR (881bp size). Using the VP7 forward and reverse primers, the partial VP7 gene was amplified in the first round. The amplicons of the VP7 first round PCR were used as the template for the VP7 second round PCR. The respective ‘G’ type was determined by using individual primers (VP7-R, G1, G2, G3, G4, G8, G9, G10, G12)^7, 8^.

Similarly, cDNA was used as a template for the VP4 first round PCR (872bp size). Using con2 and con3 primers, the partial VP4 gene was amplified in the first round. The amplicons of first round VP4 PCR were used as template for the VP4 second round PCR. The respective ‘P’ genotype was determined by using individual primers (con3, P[4], P[6], P[8] , P[9] , P[10] and P[11])^8, 9^.

### Agarose gel electrophoresis

Amplicons obtained from the second round PCR of both ‘G’ and ‘P’ types were resolved in 2% agarose gel electrophoresis in Tris acetic-acid EDTA buffer (1 M, pH 8.6). Individual genotypes were identified by their respective base pair lengths as observed in ultraviolet gel documentation system (BIORAD, Life Science, India).

### Sequencing

First round amplicons of three G12 RV strains were chosen for sequencing using ABI Prism Big Dye Terminator cycle sequencing ready reaction kit (Applied Bio-systems, USA). The sequences were resolved using an automated DNA sequencer (ABI Prism 310 genetic analyser, Applied Bio systems, USA). Access to sequencher software (Gene Codes Corporation, version 5.4.6, accessible at http://www.genecodes.com) was provided by CDC, USA and was used to read and analyse the sequences.

### Phylogenetic analysis

Three study sequences of G12 were submitted to GenBank and accession numbers were obtained (MW393832-MW393834).

Phylogenetic analysis of RV sequences were carried out using MEGA X software^10^. The evolutionary distances were inferred by Tamura Nei method^11^. In order to examine diversity, a dendrogram was constructed (maximum likelihood method) using the G12 sequences and the G10 sequences of RV strains ((MH981262, MH981263 (Feb-2016)), (MH981268-MH981276 (May-2016)), MH981279 (Dec-2015) previously isolated from our tertiary care centre.

### Statistical analysis

Statistical analysis was performed using IBM SPSS Statistics, Version 28.0.0.0. Chi-square test of significance and student's t-test were performed to analyze the data.

### Ethical approval

This study was approved by the Institutional ethics committee (IEC-NI/16/AUG/55/57).

## Results

### Prevalent genotypes

A total of 523 neonates were enrolled in the study. Of these, RV was detected in (178/523) 34% of the sample population. Of the 178 samples that tested positive for RV antigen, 169 samples were genotyped for VP7 and VP4 genes. Genotyping could not be performed in the remaining 9 samples due to insufficient sample amount. Of the 169 samples, the most prevalent G and P type combinations were G12P[11] (44.4%) and G10P[11] (42.6%). Other genotypes identified in the sample population included G3P[8] (2.9%) and G10G12P[11] (10.1%).

**Figure 1:**
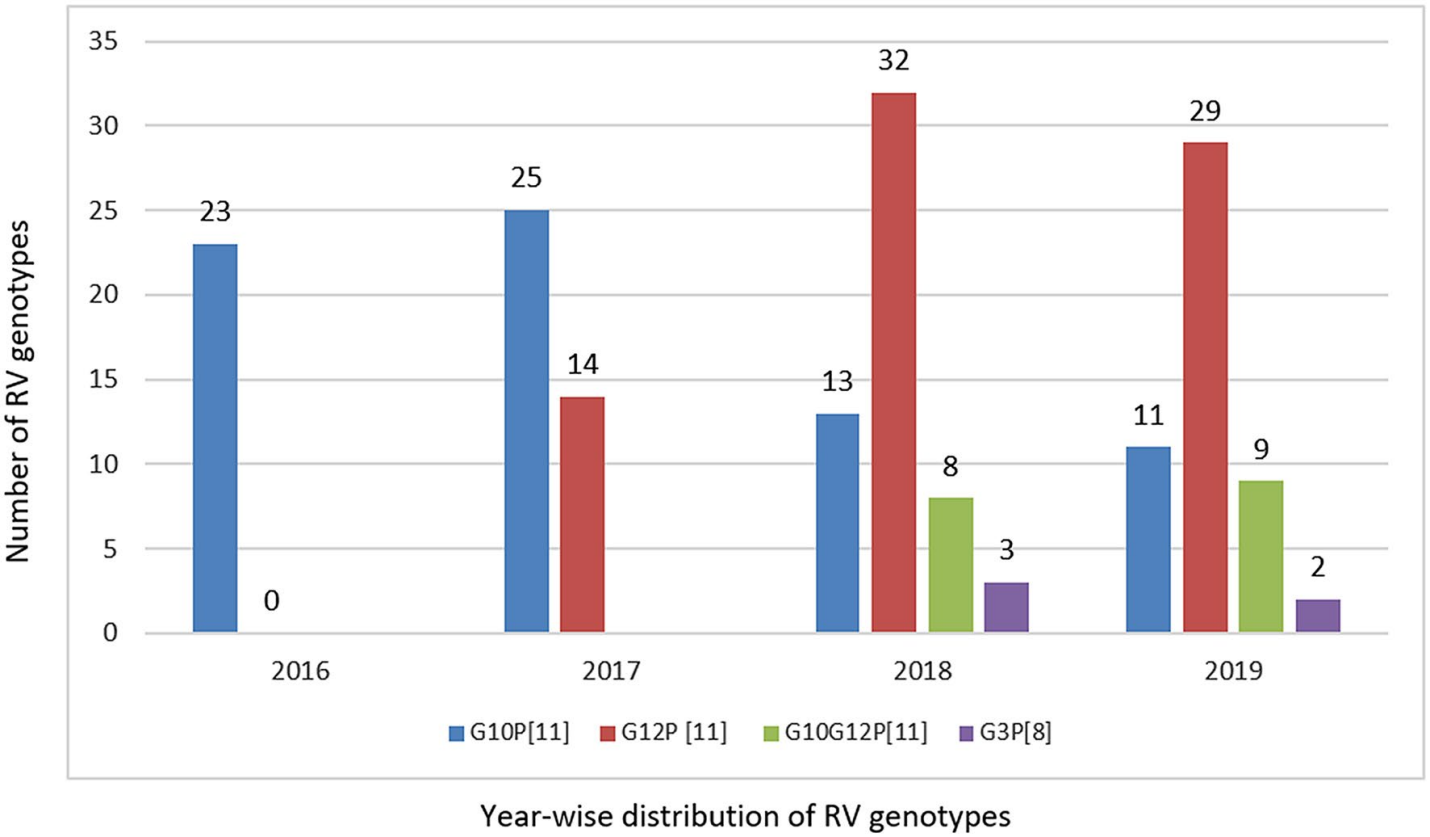
Year wise distribution of RV genotypes among neonates. This is a clear decline in G10P[11] in the past 3 years (2017–2019). G12P[11] detection rate was increased during the year 2017–2019.

The annual distribution of circulating genotypes among the hospitalized neonates were analysed (Fig. 1). In 2016, 89 samples were screened for RV and 25.8% (23/89) tested positive for RV antigen. Of these, G10P[11] was observed to be the only circulating genotype. In 2017, RV antigen was detected in 33.9% (39/115) of stool samples. Of these G10P[11] was detected in 64.1% and G12P[11] was first observed (35.9%) in our study population. Between 2018 and 2019, multiple genotypes such as G10P[11], G12P[11], G10G12P[11] and G3P[8] co-circulated in the study population. The incidence of G12P[11] increased from 35.9% in 2017 to 56.8% in 2019.

### Clinical correlates of genotypes

We correlated the circulating genotypes with clinical presentation (Table 1). GI manifestations (feeding intolerance (44.2%), abdominal distension (28.5%), gastroesophageal reflux (4.7%) of both predominant genotypes G12P[11] and G10P[11] appear to be similar. Vomiting (1.3%) and NEC (0.6%) was documented among participants with G12P[11]. The association between three non-GI manifestations (respiratory distress syndrome, apnoea of prematurity and seizures) and RV genotypes were found to not be significant. When compared with G10P[11], respiratory distress syndrome, apnoea of prematurity and seizures were observed at higher, but not significantly different frequencies among G12P[11] infected neonates (Table 1).

**Table1:** Clinical conditions of RV infected neonates with RV predominant strain.

Clinical signs	G12P[11] (n = 75) (%)	G10P[11] (n = 72) (%)	p value
**Gastrointestinal manifestation**			
Vomiting	2 (2.6)	0	NA
Abdominal distension	22 (29.3)	20 (27.7)	0.83
Feeding intolerance	34 (45.3)	31 (43)	0.78
Necrotising Enterocolitis (NEC)	1 (1.3)	0	NA
Gastroesophageal reflux	4 (5.3)	3 (4.1)	0.73
Neonatal cholestasis	1 (1.3)	1 (1.3)	0.97
Diarrhoea	0	0	NA
**Non-GI manifestation**			
Respiratory distress syndrome	13 (17.3)	9 (12.5)	0.41
Transient tachypnoea	3 (4)	2 (2.7)	0.68
Apnoea of prematurity	9 (12)	3 (4.1)	0.15
**Metabolic manifestation**			
Hypoglycaemia	3 (4)	1 (1.3)	0.33
Hyponatremia	1 (1.3)	3 (4.1)	0.29
Hyperbilirubinemia	5 (6.6)	1 (1.3)	0.10
**CNS manifestation**			
Seizure	9 (12)	5 (6.9)	0.29
**Other manifestation**			
Sepsis	3 (4)	2 (2.7)	0.68

Pearson’s Chi Square (χ^2^): 9.120, n = 147, α-0.05, one tailed, Cumulative p-value: 0.764.

NA-Not applicable, Clinical correlation of leading genotypes during study period (Sep 2016–Nov 2019) with GI manifestation and non-GI manifestation.

Neonates admitted with hypoglycaemia, hyperbilirubinemia, suspected sepsis, hyponatremia and seizure disorders (review of medical records) also shed RV. *Klebsiella pneumoniae, Salmonella typhi* and *Escherichia coli* were observed among three neonates enrolled in the study.

### Characteristics of study participants

Of the 523 neonates, 266 (50.9%) were admitted in the low-cost facility, and others were admitted to the high-cost facility. A total of 310 (59.3%) stool samples were collected from the neonatal intensive care unit and 213 (40.7%) from neonatal nursery. Most babies were born by lower segment cesarean section (LSCS) (74.7%) as the hospital is a referral centre for management of pregnancy with clinical indication of cesarean section. Male neonates (54.3%, 284/523) outnumbered their female counterparts (45.7%, 239/523) in admissions.

RV antigen was shed in 38.7 % (103/266) of neonates admitted to the low-cost facility when compared to neonates admitted to the high-cost facility (29.2%). The difference in detection was significantly higher among babies admitted to the low-cost facility (p < 0.05). Similarly, 37.7% (117/310) of neonates admitted in the neonatal ICU shed RV in stools in contrast to babies admitted in the neonatal nursery, of whom only 28.6% (61/213) shed RV as shown in Table 2. Babies admitted to the neonatal ICU shed significantly more RV than babies in the neonatal nursery (p < 0.05).

**Table 2:** Demographic profile and characteristics of study participants among RV positive and negative neonates.

	RV negative n = 345 (%)	RV positive n = 178 (%)	p value
**Gender**			
Male	183 (53)	101 (56.7)	0.42
Female	162 (47)	77 (43.3)	
**Hospital facility**			
Low-cost facility	163 (51.4)	103 (57.9)	0.02
High-cost facility	182 (48.6)	75 (42.1)	
**Hospital settings**			
Neonatal Intensive care Unit	193 (55.9)	117 (65.7)	0.03
Neonatal nursery	152 (44.1)	61 (34.3)	
**Birth weight**			
Normal 2.5 kg–> 4kg	152 (44.1)	86 (48.3)	0.03
Low birth weight 1 kg–< 2.5kg	166 (48.1)	88 (49.4)	
Extremely low birth weight < 1 kg	27 (7.8)	4 (2.3)	
**Gestational age**			
Post term (> 39 weeks)	12 (3.4)	5 (2.8)	0.01
Term (37–< 39 weeks)	36 (10.4)	23 (12.9)	
Moderate or late preterm (34–< 37 weeks)	103 (29.8)	74 (41.5)	
Very preterm (28<34 weeks)	167 (48.4)	71 (39.8)	
Extremely premature (22-<28 weeks)	27 (7.8)	5 (2.8)	
**Mode of delivery**			
Lower segment caesarean section	253 (73.3)	138 (77.5)	0.24
Normal vaginal delivery	85 (24.6)	40 (22.5)	
Forceps	3 (0.9)	0	
Vacuum	4 (1.2)	0	
**Type of feeding**			
Direct breast feeding	48 (13.9)	24 (13.5)	0.89
DBF + formula feeding	56 (16.2)	27 (15.2)	
Formula feeding	136 (39.4)	67 (37.6)	
TPN + formula feeding	105 (30.4)	60 (33.7)	

Pearson’s Chi Square (χ^2^):34.26, n = 523, α-0.05, one-tailed, Cumulative p-value: 0.034.

*TPN* total parenteral nutrition, *DBF* direct breast feeding.

RV was observed in 35.6% (101/284) of male neonates, and in 32.2% (77/239) of female neonates. RV antigen was detected in 35.3% of (138/391) neonates delivered by LSCS and 30.3% (40/132) of vaginally delivered neonates. Gender and mode of delivery did not seem to influence RV infection (Table 2).

RV antigen was detected in 33.3% (24/72) of neonates who were exclusively breastfed, 32.5% (27/83) of neonates who were breastfed and on formula, 33% (67/203) of babies who were only on formula and 36.3% (60/165) of babies on TPN (Total Parental Nutrition) with formula feeds. Feeding habits did not affect RV shedding.

The possibility of gestational age as a risk factor of RV infection was analysed among RV-infected asymptomatic neonates (Table 2). RV infection was significantly higher (p < 0.05) among term neonates 38.9% (23/59) and late preterm neonates 41.8% (74/177). The detection of RV among post term 29.4% (5/17), very preterm 29.8% (71/238) and extremely pre-term 15.6% (5/32) babies was comparatively lower within the study population. RV infection was similar among normal birth weight babies (36.1%) and low birth weight babies (34.6%). RV detection was low (12.9%) among extremely low birth weight babies.

**Figure 2:**
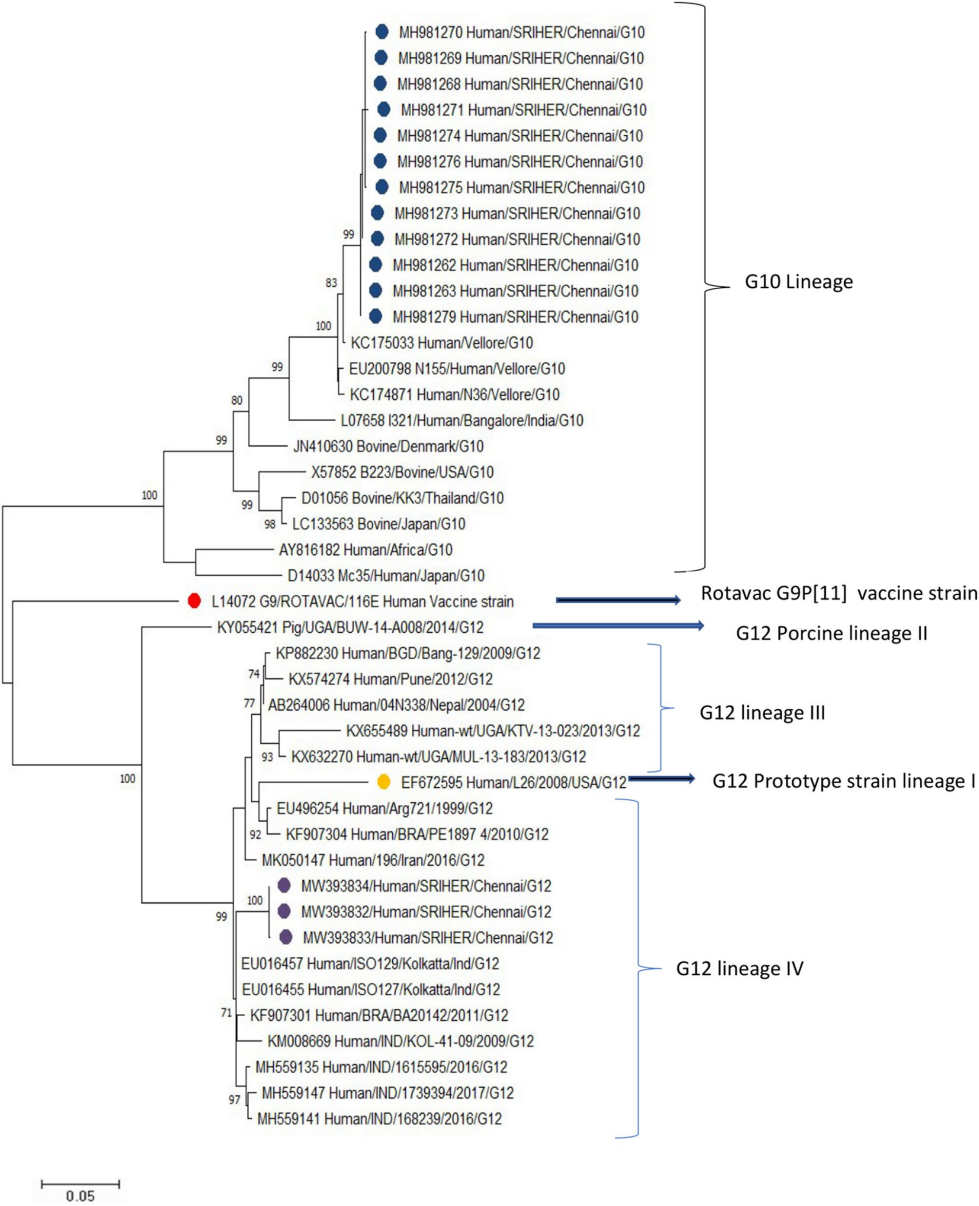
Molecular phylogenetic analysis by maximum likelihood method. Phylogenetic analysis of VP7 gene of G12 and G10 RV strains. The study strains G12 are indicated in purple circle, and G10 study strains are shown in blue circle. The phylogenetic tree was constructed using the Maximum Likelihood method and Kimura 2-parameter model using MEGA-X. Bootstrap values (1000 replicates) less than 70% are not shown. Scale bar indicates the evolutionary distance (nucleotide substitution per site).

G12 sequences from our study clustered with each other with a nucleotide identity of 99.87% to 100%. The G12 strains clustered with the Kolkata strains with 97.3 to 98% nucleotide identity followed by the Pune strain with 96.7% to 97% identity. The G12 strains also clustered with human strains from different parts of the world such as Brazil (94.4–97.1%), Argentina (94.8%), Iran (96%) and Uganda (90-94.5%) (Fig. 2).

G10 sequences from our study clustered with each other with a nucleotide identity of 99.6% to 100%. Our study sequences closely clustered to Vellore strains with a nucleotide identity of 97.4% to 98.2%. However, it was distant from Bangalore strain (I321) with a nucleotide identity of only 91.1%. Our strains show around 90% nucleotide identity with bovine strains circulating in different parts of the world like Thailand, Japan and USA (Fig. 2).

## Discussion

RV genotypes previously encountered among neonates include G4P[6] from South Africa^4^, G8P[6] from Malawi^12^, G4P[8] from Greece^13^, G12P[6] from India (Delhi)^14^, G9P[11]from India (Delhi)^15^ and G10P[11] from India (Bangalore)^16^. It is evident that different genotypes are present in different parts of the world. Between 1988 and 2016, G10P[11] was the single circulating genotype in the neonatal nursery and NICU for several years in South India^17^. The lack of diversity in the circulating genotypes in the neonatal nursery and neonatal ICU was considered a hallmark of neonatal RV infection. Neonatal strains are distinct and are known to be endemic in the neonatal unit^18^. The reassortant of two human-bovine reassortants, G9P[11] and G9P[6] strains among neonates were first described in 1985^19^. Subsequently, a mixed genotype G9P[6] P[11] has been reported^15^.

G10P[11] was first detected in 1988 and is unique among neonates in India. G10P[11] has been associated with nosocomial outbreaks in the neonatal ICU and nursery in Bangalore between 1988 and 1999 and in Vellore between 2006 and 2007^5, 16^. G10P[11] I321 strain causes both symptomatic and asymptomatic RV infection among neonates^16^. G10P[11] was also documented as the circulating strain in the hospital units investigated between 2014 and 2016^17^.

The co-circulation of G10P[11] and G12P[11] was initially observed in 2017, with G10P[11] being the predominant strain. However, there has been a steady increase in the circulation of G12P[11]. We also documented the emergence of G12P[11] between 2017 and 2019. The frequency of G12P[11] notably increased from 35.9 to 56.8% between 2017 and 2019 making it the predominant circulating genotype in the neonatal ICU and nursery. The emergence of G12P[11] among neonates has been previously reported in Pune and found to be 24.3%^20^. In 2014, G12P[11] first emerged in Kolkata, India in children under 5 years^21^. Little is known about whether G12P[11] circulates worldwide.

Phylogenetic analysis of G12 strains shows 88.5% nucleotide identity with prototype strain L26 in lineage I which was first reported in the Philippines in 1987. The G12 genotype origin is unknown, although pigs are believed to be its primary host^20^. Majority of the study strains belonged to lineage IV with P[9], P[8], and P[11] types. The G12 strain has previously been reported in children with a combination of P[8], P[6] and P[4] types.

G3P[8] was first observed in 2015 among neonates in Greece. In our centre, G3P[8] was documented as an emerging strain in 2018. Within neonates, the distribution of G3P[8] observed was 5.8% (2018) and 3.7% (2019). For the purpose of analysis, GI manifestations were considered as symptomatic and non-GI manifestations were considered to be asymptomatic^5^. Of the five G3P[8] genotypes in this study, GI manifestations (20%) were observed with only one genotype. Non-GI manifestations were observed among the other four genotypes. Recently, G3P[8] has been increasingly reported in children under 5 years. In 2014, G3P[8] represented a small proportion of rotaviral gastroenteritis (1.9%) within children under five years of age in India^22^.

This is the first study to document the G10G12P[11] mixed genotypes among neonates. P[11] genotype (known to be bovine in origin) in a combination with G9 and G10 was observed among neonates in India. The G9P[11] and G10P[11] genotypes were found only in India and have been identified as natural human bovine reassortant rotavirus^23^. A combination of P[11] with either G10 or G12 may co-infect an individual and provide opportunities for reassortment in infected individuals.

This study also documents the emergence of G12P[11], mixed genotype G10G12P[11] and G3P[8] in the neonatal nursery and NICU. A study from Greece in 2013 has documented four different genotypes among neonates. Between 2009 and 2013, multiple genotypes (G4P[8], G12P[8], G1P[8], G3P[8]) among neonates have been documented in Greece^13^. However, prior to 2009, there are no data on RV genotypes circulating in Greece. Whether the emergence of multiple genotypes is a sporadic occurrence or worldwide phenomenon needs to be further explored.

G10P[11] was the primary circulating strain among neonates for nearly three decades. G12P[11] and G3P[8] were believed to infect only children under five years. However, this study has also documented G12P[11] and G3P[8] among neonates.

Does the emergence of multiple genotypes and the potential of G12P[11] and G3P[8] to infect both children under five years and neonates constitute a paradigm shift? The term ‘paradigm shift’ was introduced by Thomas Kuhn, one of the most eminent philosophers of the twentieth century. He proposed two kinds of scientific change: one which constitutes standard science characterized by incremental changes, and the other a scientific revolution that permits the differentiation of periods of standard science. He stated that scientific revolutions that do not occur due to incremental changes can be characterized as a paradigm shift^24^. Further studies from other centers will assist in determining whether the emergence or shift to multiple genotypes after several decades does constitute a paradigm shift.

The rationale for an evolving G type while the P type relatively stays the same could possibly be based on host and/or environmental factors. This should be further explored. With the current dearth in data, continuous extensive surveillance should be undertaken to study the divergence of RV in neonates on a global scale.

Among neonates, RV is frequently associated with both non-gastrointestinal (asymptomatic) and gastrointestinal manifestations (symptomatic). In our study, 36.9 % of neonates had gastrointestinal manifestations, the most common of which were feeding intolerance (44.2%) followed by abdominal distension (28.5%). These manifestations were noted to be associated with G12P[11] and G10P[11]. In Vellore, feeding intolerance was documented to be 32.7% and in Greece, it was found to be 39.6%^5, 13^. In Vellore, a genotype-specific analysis found no difference between genotypes and GI/Non-GI manifestations^5^. RV infections in neonates presenting with a host of other conditions such as respiratory distress syndrome and apnoea of prematurity could be attributed more to G12P[11] than G10P[11] genotypes. Since G10P[11] was the only circulating genotype for nearly three decades, the need to correlate the genotype with clinical manifestation did not arise. However, with the current emergence of diversity in circulating genotypes, it is important to correlate clinical manifestation with genotypes to ascertain any preferential association.

Neonatal RV strains have been employed as vaccine/vaccine candidates. Asymptomatic neonatal strain (116E) of G9P[11] was used to manufacture Rotavac vaccine in India^25^. Similarly, in 1975-1976, RV3-BB G3P[6], another asymptomatic neonatal strain was documented in Victoria and is being explored as a vaccine candidate in Australia^26^. The continuous surveillance of local strain diversity is important to assess and consider the strain variability in the design of new vaccine candidate.

Neonates infected with asymptomatic strains were subsequently protected from severe disease in early childhood^19, 27^. The cross protection against RV has been documented irrespective of the predominantly circulating genotype at the time^28^. Velazquez et al. reported protection among 87% of neonates when re-exposed to RV^29^. The protection acquired through wild-type infection is similar to that acquired via vaccination. However, a study from South India documented the absence of cross-protection among children under 5 years who had previous neonatal RV infection^30^.

RV can cause nosocomial infection and is likely to spread in NICU and neonatal nurseries^13^. The first RV nosocomial outbreak was reported in 1975 among neonates in London^31^. As nosocomial transmission is likely to happen 48 hours after admission, we enrolled neonates and collected samples at the respective time. Among asymptomatic neonates, the data from our study may possibly represent colonization of the gut and nosocomial transmission.

The neonates enrolled in our study were admitted in the different facilities, one at a low-cost facility that caters to a lower socioeconomic group and the other, a high-cost facility. The shedding of RV antigen was found to be significantly higher in the low-cost facility. Both facilities are manned by staff trained in basic infection control. These findings suggest that neonates belonging to the lower socioeconomic strata are more prone to RV infection.

There are reports of gender disparity in the acquisition of RV among children^28^. However, in our study, we did not observe any association between RV infection and gender. Neonates delivered vaginally appeared to be more prone to RV infection^5^. However, in our study, there was no noticeable difference in acquisition between neonates born by lower segment caesarean section (LSCS) versus vaginal delivery. The feeding habits did not appear to influence shedding of RV.

A limitation of this study is that the comparison of the VP4 gene of G10P[11] and G12P[11] strains could not be undertaken to determine if the same variant is present in both G10P[11] and G12P[11]. While it is interesting that G12P[11] strains are circulating, we consider that whole genome sequencing will probably provide further information on the P[11] variant among hospitalized neonates.

There are a few follow-up studies that determine the outcome of RV neonatal infection. Longitudinal follow-up studies will assist in determining whether colonization with RV will protect against severe RV disease or possibly cause complications. Additionally, there are host and nutrition related factor that may contribute to the outcome of RV infection.

## Conclusion

Until 2016, G10P[11] was reported to be the only circulating genotype among hospitalized neonates in South India. We documented the preponderance of an emerging G12P[11] genotype among neonates. Following the emergence of multiple genotypes, there is a need to study prospectively whether this emergence will confer protection against future RV infection. The possible accidentality of the relationship observed in RV-positive neonates with non-GI manifestations needs to be further explored. With increasing diversity within circulating genotypes, the surveillance of RV to identify emerging strains is important and establish the road ahead in managing RV infection (Supplementary Fig. S1).

## Supplementary Information

Supplementary Figure S1.
