# Supplementary figures and images for: A shift in circulating rotaviral genotypes among hospitalized neonates
Source: Sci Rep. 2022 Feb 18;12:2842. doi: 10.1038/s41598-022-06506-y (PMC8857175; doi:10.1038/s41598-022-06506-y)

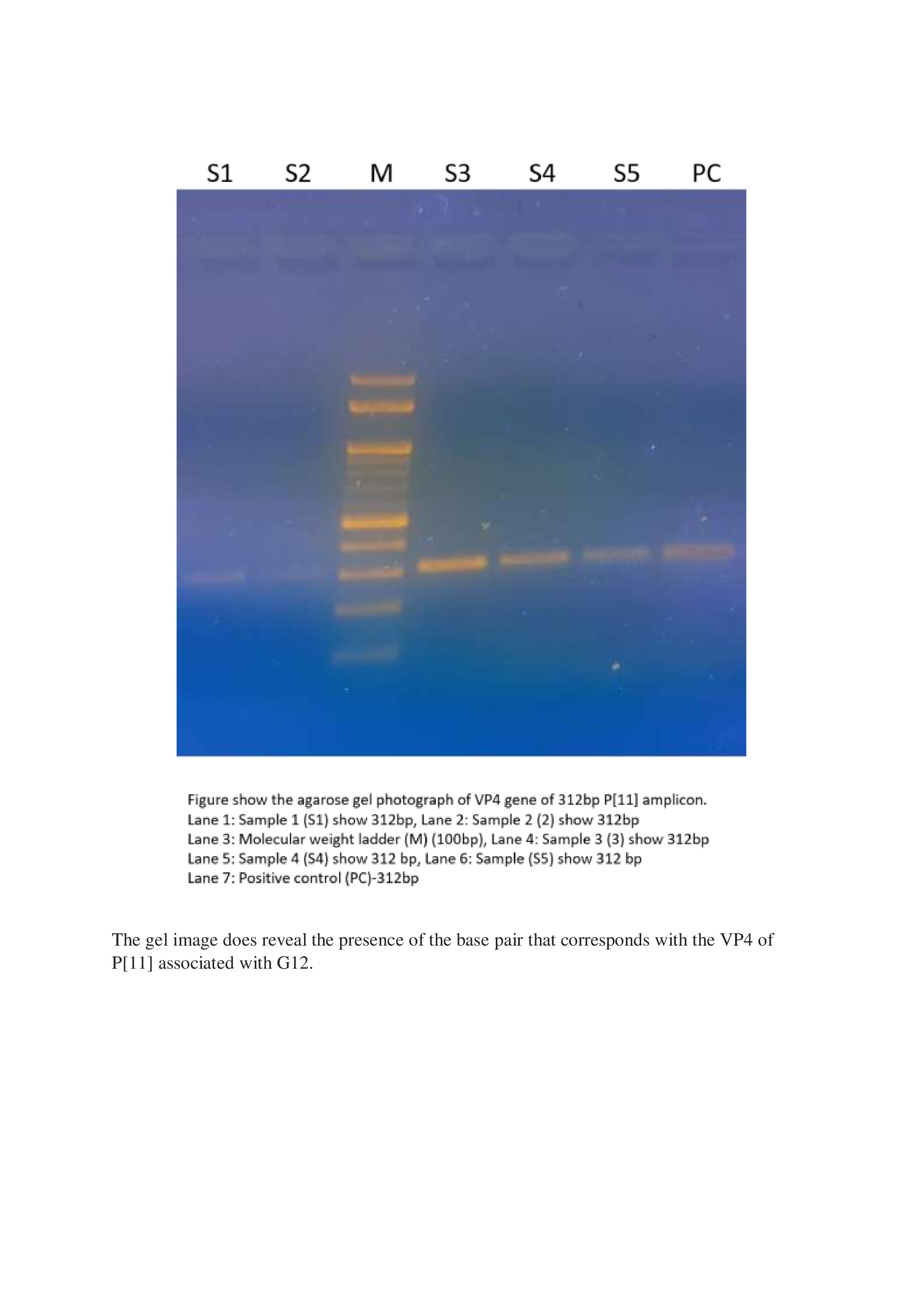

Supplement: Supplementary file 1 — Supplementary Figure S1. [file 41598_2022_6506_MOESM1_ESM.tiff]
